# Evaluation of Sensitivity and Specificity Performance of Elecsys HTLV-I/II Assay in a Multicenter Study in Europe and Japan

**DOI:** 10.1128/JCM.00169-17

**Published:** 2017-06-23

**Authors:** Syria Laperche, Silvia Sauleda, Maria Piron, Annelies Mühlbacher, Harald Schennach, Volkmar Schottstedt, Lucinda Queirós, Naoki Uno, Katsunori Yanagihara, Roland Imdahl, Ariann Hey, Markus Klinkicht, Walter Melchior, Peter Muench, Toshiki Watanabe

**Affiliations:** a Institut National de la Transfusion Sanguine (INTS), Département des agents transmissibles par le sang, Centre National de Référence Risques Infectieux Transfusionnels, Paris, France; b Banc de Sang i Teixits, Barcelona, Spain; c Central Institute for Blood Transfusion and Immunology, University Hospital, Innsbruck, Austria; d DRK Blutspendedienst West Central Laboratory, Hagen, Germany; e Instituto Português do Sangue e da Transplantação, Porto, Portugal; f Department of Laboratory Medicine, Nagasaki University Hospital, Nagasaki, Japan; g Labor Schottdorf MVZ, Augsburg, Germany; h Roche Diagnostics, Penzberg, Germany; i Graduate School of Frontier Sciences, University of Tokyo, Tokyo, Japan; j Department of Advanced Medical Innovation, St. Marianna University Graduate School of Medicine, Kanagawa, Japan; Memorial Sloan Kettering Cancer Center

**Keywords:** Elecsys, HTLV-1, HTLV-2, blood screening, diagnosis, electrochemiluminescence, immunoassays, sensitivity, specificity

## Abstract

Screening of blood for human T-cell lymphotropic virus type 1 and type 2 (HTLV-1 and -2, respectively) is important to diagnose and prevent infection and ensure the safety of blood supplies. The Elecsys HTLV-I/II assay is a newly developed, electrochemiluminescence screening assay for the detection of HTLV-1/2 infection. The sensitivity and specificity of the Elecsys HTLV-I/II assay were determined using well-characterized HTLV-1/2-positive serum and plasma samples and routine diagnostic and blood donor samples expected to be HTLV negative, respectively. These results were compared with those for at least one of the following CE-marked assays at seven independent laboratories and the Roche Diagnostics facility in Penzberg, Germany: Abbott Architect rHTLV-I/II, Ortho Avioq HTLV-I/II Microelisa system, Abbott Prism HTLV-I/HTLV-II, and DiaSorin Murex HTLV I+II. Fujirebio INNO-LIA HTLV-I/II Score was used as a confirmatory assay. The Elecsys HTLV-I/II, Abbott Architect rHTLV-I/II, and Abbott Prism HTLV-I/HTLV-II assays detected all HTLV-1/2-positive samples (sensitivity, 100%). Sensitivity for Ortho Avioq HTLV-I/II was 98.63%. The Elecsys HTLV-I/II assay had a specificity of 99.95% in blood donor samples, which was comparable to results for the other assays (range, 99.91 to 100%). In routine diagnostic samples, the specificity of the Elecsys HTLV-I/II assay was 99.83%, compared with 99.70% for Abbott Architect rHTLV-I/II. Specificity for the Elecsys HTLV-I/II assay in potentially cross-reactive samples was 100%, compared with 99.0% for Ortho Avioq HTLV-I/II and 99.2% for DiaSorin Murex HTLV I+II. The Elecsys HTLV-I/II assay has the sensitivity and specificity to support its use as a routine screening assay for detecting HTLV infection.

## INTRODUCTION

Human T-cell lymphotropic virus (HTLV) is a cell-associated retrovirus, and four types have been identified to date. Type 1 (HTLV-1) is the only type that has been linked conclusively to disease. Infection with HTLV-1 is associated with the development of adult T-cell leukemia/lymphoma (ATL) and HTLV-1-associated myelopathy/tropical spastic paraparesis (HAM/TSP), with the former occurring in up to 5% and the latter in up to 3% of infected individuals (1, 2). HTLV-1 infection is also linked to a number of inflammatory conditions other than HAM/TSP (3). In contrast to HTLV-1, HTLV-2 has not been proven to cause disease (4). However, HTLV-2 infection has been associated with increased lymphocyte and platelet counts (5), an increased overall cancer mortality (6), sporadic reports of myelopathy (7), and a slightly increased risk of bacterial infections, particularly of the chest and bladder/kidney (8). The potential for HTLV-3 and HTLV-4 to cause disease is currently unknown (9).

HTLV-1 and HTLV-2 are the most prevalent types, but their global distribution is unusual and their prevalence is poorly understood. The most recent estimate suggests that 5 to 10 million people worldwide are infected with HTLV-1, but the actual number of infected individuals is likely to be much higher (10). HTLV-1 is highly endemic in the southwestern part of Japan; parts of sub-Saharan Africa, South America, and the Caribbean; and certain foci in the Middle East and Australo-Melanesia (10). In comparison, HTLV-2 is prevalent in indigenous populations of Africa, native inhabitants of Central and South America, and intravenous drug users in Europe and the United States (11, 12).

HTLV is transmitted through sexual intercourse, blood transfusions, organ transplantation, and needle reuse or from mother to child, predominantly during breastfeeding (13, 14). HTLV has also been shown to be acquired by humans through a bite from a simian T-lymphotropic virus type 1 (STLV-1)-infected nonhuman primate (15, 16). In the absence of a preventive vaccine and with limited treatment options for HTLV-related diseases, the focus is on prevention of viral transmission by diagnosing infected individuals and providing appropriate education. However, HTLV-1/2 infection remains underdiagnosed, partly because the majority of infections are asymptomatic.

Laboratory screening for HTLV-1/2 infection has become routine for blood donors in developed and some developing countries where the virus is endemic or where blood donors are considered to be at risk of infection (17, 18, 29). In low-seroprevalence populations, the positive predictive power of any assay is low and most positive results are false positives (4). For this reason, samples testing positive with screening tests should be tested with confirmatory assays. The development of screening assays with outstanding sensitivity and specificity would be valuable in improving HTLV-1/2 detection in the diagnostic setting, in the screening of blood donors, and for epidemiologic studies.

The Elecsys HTLV-I/II assay is a newly launched, double-antigen sandwich screening assay. The assay detects the immune response against the gp21 and p24 antigens (19). The immune response to p24 is one of the earliest detectable responses in HTLV-1/2 infection, and specific inclusion of this antigen in the Elecsys HTLV-I/II assay ensures that early infections are not missed (20). The objective of this study was to evaluate the clinical sensitivity and specificity of the Elecsys HTLV-I/II assay using well-characterized HTLV-1/2-positive serum and plasma samples and routine diagnostic and blood donor samples expected to be HTLV negative, respectively, in a head-to-head comparison with other commercially available, CE-marked HTLV-1/2 screening assays, and to assess its specificity using potentially cross-reactive samples.

## RESULTS

### Sensitivity.

The overall sensitivity of each assay is shown in Table 1. All samples were correctly identified as positive for HTLV-1/2 antibody by the Elecsys HTLV-I/II, Abbott Architect rHTLV-I/II, and Abbott Prism HTLV-I/HTLV-II assays (small sample size, *n* = 10) (sensitivity, 100%), while Ortho Avioq HTLV-I/II missed three HTLV-1-positive samples (sensitivity, 98.63%).

**TABLE 1:** Overall sensitivity analysis for the Elecsys HTLV-I/II assay and comparator assays by geographic origin of samples

Cohort (geographic origin of samples)^*a*^	Elecsys HTLV-I/II		Abbott Architect rHTLV-I/II		Ortho Avioq HTLV-I/II		Abbott Prism HTLV-I/HTLV-II	
	No. tested	% sensitivity	No. tested	% sensitivity	No. tested	% sensitivity	No. tested	% sensitivity
Caribbean	97	100	97	100				
South America	134	100	134	100				
Europe/Middle East	236	100	17	100	219	98.63^*b*^		
Africa	3	100	3	100				
USA	259	100	249	100			10	100
Japan	420	100	420	100				
Overall sensitivity	1,149	100	920	100	219	98.63	10	100

a Countries within regions: Caribbean—Guadeloupe and Martinique; South America—Bolivia, Chile, Colombia, Ecuador, French Guiana, Honduras, and Peru; Europe/Middle East—France, Germany, Iran, Romania, and Spain; Africa—Ivory Coast, Morocco, and Senegal.

b False-negative results were reported for three precharacterized HTLV-1-positive samples at the Paris laboratory.

### Specificity.

The overall specificity in blood donor samples was 99.91% for the Abbott Architect rHTLV-I/II, 99.95% for the Elecsys HTLV-I/II (based on results for serum and plasma samples) and Ortho Avioq HTLV-I/II assays, and 100% for Abbott Prism HTLV-I/HTLV-II (Table 2). One sample from the Barcelona center was excluded from the specificity calculations, having given the following results: Elecsys HTLV-I/II reactive, Abbott Architect rHTLV-I/II nonreactive, and Fujirebio INNO-LIA HTLV-I/II score reactive (“env gp21 I/II” [“1+”], “env gp46 I/II,” “env gp46-I” [“+/−”]). No clarification by nucleic acid technology (NAT) testing or by a sequential bleed was possible due to anonymization of samples. The specificity results for the assays in blood donor samples at the individual laboratories are shown in Table S1 in the supplemental material.

**TABLE 2:** Overall specificity analysis for the Elecsys HTLV-I/II assay and comparator assays in blood donor samples

Parameter^*b*^	Elecsys HTLV-I/II			Abbott Architect rHTLV-I/II serum	Abbott Prism HTLV-I/HTLV-II serum	Ortho Avioq HTLV-I/II serum
	Total	Serum	Plasma			
No. of samples						
Total	11,574^*a*^	9,550^*a*^	2,024	5,737^*a*^	3,813	2,024
Negative	11,568	9,546	2,022	5,734	3,812	2,022
With ≥1 s/co						
IRpos	9	8	1	6	5	3
IRfpos	6	6	0	5	5	2
RRpos	9	8	1	6	0	2
RRfpos	6	6	0	5	0	1
No. of samples with HTLV-1/2 immunoblot assay result/total no. tested						
Positive	0/9	0/8	0/1	0/6	0/0	0/3
Indeterminate	3/9	2/8	1/1	1/6	0/0	1/3
Negative	6/9	6/8	0/1	5/6	0/0	2/3
% specificity IR ≥ 1 s/co	99.95	99.94	100	99.91	99.87	99.90
95% confidence limit (two-sided, IR ≥ 1)	99.89–99.98	99.86–99.98	99.82–100	99.80–99.97	99.69–99.96	99.64–99.99
% specificity RR ≥ 1 s/co	99.95	99.94	100	99.91	100	99.95
95% confidence limit (two-sided, RR ≥ 1)	99.89–99.98	99.86–99.98	99.82–100	99.80–99.97	99.90–100	99.72–100

a One sample excluded from specificity calculations: Elecsys HTLV-I/II reactive; Abbott Architect rHTLV-I/II nonreactive; Fujirebio INNO-LIA HTLV I/II Score reactive, “env gp21 1/2” (“1+”), “env gp46 1/2,” “env gp46-I” (“+/−”). No clarification by nucleic acid technology (NAT) testing or by a sequential bleed was possible due to anonymization of samples.

b Total, number of analyzed samples; negative, number of true-negative samples (excluding indeterminate, positive, and false-negative samples); IR, initially reactive; IRpos, initially reactive sample, including true-positive samples; IRfpos, initially reactive sample, not including true-positive samples; RR, repeatedly reactive; RRpos, repeatedly reactive sample, including true-positive samples; RRfpos, repeatedly reactive sample, not including true-positive samples; s/co, signal/cutoff ratio.

The overall specificity in routine diagnostic samples was 99.70% for Abbott Architect rHTLV-I/II (the only comparator assay used in this part of the study) and 99.83% for the Elecsys HTLV-I/II assay (Table 3). The specificity results for the assays in routine diagnostic samples at the individual laboratories are shown in Table S2.

**TABLE 3:** Overall specificity analysis for the Elecsys HTLV-I/II and Abbott Architect rHTLV-I/II assays in routine diagnostic samples

Parameter^*a*^	Elecsys HTLV-I/II serum	Abbott Architect rHTLV-I/II serum
No. of samples		
Total	2,399	2,399
Negative	2,336	2,336
With ≥1 s/co		
IRpos	66	68
IRfpos	4	7
RRpos	66	68
RRfpos	4	7
No. of samples with HTLV-1/2 immunoblot assay result/total no. tested		
Positive	59/66	59/68
Indeterminate	3/66	2/68
Negative	4/66	7/68
% specificity IR ≥ 1 s/co	99.83	99.70
95% confidence limit (two-sided, IR ≥ 1)	99.56–99.95	99.38–99.88
% specificity RR ≥ 1 s/co	99.83	99.70
95% confidence limit (two-sided, RR ≥ 1)	99.56–99.95	99.38–99.88

a Total, number of analyzed samples; negative, number of true-negative samples (excluding indeterminate, positive, and false-negative samples); IR, initially reactive; IRpos, initially reactive sample, including true-positive samples; IRfpos, initially reactive sample, not including true-positive samples; RR, repeatedly reactive; RRpos, repeatedly reactive sample, including true-positive samples; RRfpos, repeatedly reactive sample, not including true-positive samples; s/co, signal/cutoff ratio.

There were no notable differences in specificity results across the individual laboratories.

### Cross-reactivity/interference.

The Elecsys HTLV-I/II assay was shown to be unaffected by icterus (bilirubin, ≤1,129 μmol/liter or ≤66 mg/dl), hemolysis (hemoglobin [Hb], ≤0.3 mmol/liter or ≤0.5 g/dl), lipemia (intralipid, ≤2,000 mg/dl), and biotin (≤246 nmol/liter or ≤60 ng/ml) during development (data not shown).

In the investigation of possible cross-reacting factors at the laboratories in Paris and Penzberg, no false-positive result was found with the Elecsys HTLV-I/II assay (specificity, 100%). At the Paris laboratory, the Ortho Avioq HTLV-I/II assay showed one false-positive result for a precharacterized hepatitis B virus (HBV)-positive sample (specificity, 99.0%; signal/cutoff ratio [s/co] initial reactive [IR] = 1.22; repeats = 1.22/0.64). At the Penzberg laboratory, the DiaSorin Murex HTLV I+II assay showed one false-positive result for a precharacterized rubella virus-positive sample (specificity, 99.2%; s/co IR = 1.99; repeats = 1.89/0.28).

## DISCUSSION

This study demonstrates that the Elecsys HTLV-I/II assay has the sensitivity (100%) and specificity (99.95% in blood donor samples, 99.83% in routine diagnostic samples), regardless of the geographic origin of the samples, virus type, or location of the testing laboratory, to support its use as a routine screening assay worldwide.

The Abbott Architect rHTLV-I/II and Abbott Prism HTLV-I/HTLV-II assays also identified all precharacterized HTLV-1/2-positive samples correctly (sensitivity, 100%). These findings are consistent with previously reported results. In an earlier study of 406 precharacterized HTLV-1/2-positive samples, Abbott Architect rHTLV-I/II had a sensitivity of 100% (21, 22). Similarly, in a study of 714 confirmed HTLV-1/2-positive samples, a sensitivity of 100% was reported for Abbott Prism HTLV-I/HTLV-II (23). Based on 636 precharacterized HTLV-1/2-positive samples, the sensitivity of Ortho Avioq HTLV-I/II has been reported previously as 100% (24). This compares with a sensitivity of 98.63% for Ortho Avioq HTLV-I/II in the present study (three false-negative results at the Paris laboratory).

Overall, the Elecsys HTLV-I/II assay (99.95%) had specificity comparable to that of Ortho Avioq HTLV-I/II (99.95%), numerically lower specificity than Abbott Prism HTLV-I/HTLV-II (100%), and numerically higher specificity than Abbott Architect rHTLV-I/II (99.91%) when tested in blood donor samples. The specificity of the Elecsys HTLV-I/II assay (99.83%) was numerically higher than that of Abbott Architect rHTLV-I/II (99.70%) when tested in routine diagnostic samples. The confidence intervals of the specificity data for all four screening assays were overlapping, and therefore, these numerical differences were not statistically significant. All assays showed consistent specificity regardless of the location of the testing laboratory. The results for the comparator assays were consistent with previous data. In a study of 5,646 blood donor samples, Abbott Architect rHTLV-I/II had a specificity of 99.95%, compared with 99.86% when tested in 692 routine diagnostic samples (21, 22). Ortho Avioq HTLV-I/II had a specificity of 99.95% in a study of 11,415 blood donor samples (24), while Abbott Prism HTLV-I/HTLV-II had a specificity of 99.93% in a study of 21,943 blood donor samples (23).

In certain geographic regions, particularly in Africa, which represents the largest area where HTLV-1 infection is endemic, there is a wide genetic variability of HTLV-1 genotypes and a large pattern of indeterminate serology exists (25, 26). Unfortunately, the number of samples available for analysis from African regions during this study was limited. In addition, it was not possible to obtain genotypic information for specific samples due to the unavailability of whole blood in order to extract peripheral blood mononuclear cells (PBMCs). Therefore, future studies might aim to investigate the performance of HTLV screening assays in a larger series of samples from African individuals, including those with an HTLV-1 Gag-indeterminate pattern (HGIP) or new Western blot (NWB) profile, in order to assess the performance of these assays in this extremely important demographic group.

The Elecsys HTLV-I/II assay is a quick (assay time, 18 min), one-step (double-antigen sandwich) assay, and the simplicity of the methodology and speed of the assay offer clear benefits in terms of the number of samples that can be processed by a testing facility within a given time. Furthermore, the antigen combination in the Elecsys HTLV-I/II assay was not associated with any cross-reactivity in our study.

In summary, the Elecsys HTLV-I/II assay is a reliable test for screening for HTLV-I/II infection in the blood and organ donation settings and for diagnosis where there is suspicion of HTLV-associated diseases, such as ATL, HAM/TSP, and undiagnosed myelopathy.

## MATERIALS AND METHODS

### Participating laboratories and compliance.

Seven independent laboratories from six countries (Austria [Innsbruck], France [Paris], Germany [Hagen and Augsburg], Japan [Nagasaki], Portugal [Porto], and Spain [Barcelona]) and the Roche Diagnostics facility at Penzberg in Germany participated in the study (Table 4). The study was conducted in compliance with relevant directives of the European Union (EU) parliament and EU council. Ethical approval for the study was obtained from independent review bodies when required by national regulations, and waivers from ethical committees were obtained by each laboratory participating in the study, as necessary. All samples were deidentified or coded prior to use, and therefore, individual patient data were not available for this study.

**TABLE 4:** Screening assays and platform for the Elecsys HTLV-I/II assay used by each of the laboratories

Laboratory	Comparator assay(s) used	Platform for the Elecsys HTLV-I/II assay
Central Institute for Blood Transfusion and Immunology, University Hospital, Innsbruck, Austria	Abbott Architect rHTLV-I/II	Modular Analytics E170
	Ortho Avioq HTLV-I/II Microelisa system	
DRK Blutspendedienst West, Hagen, Germany	Abbott Prism HTLV-I/HTLV-II	cobas e 602
Labor Schottdorf MVZ, Augsburg, Germany	Abbott Architect rHTLV-I/II	Modular Analytics E170
Instituto Português do Sangue e da Transplantaçăo, Porto, Portugal	Abbott Architect rHTLV-I/II	cobas e 601
Banc de Sang i Teixits, Barcelona, Spain	Abbott Architect rHTLV-I/II	cobas e 411
Institut National de la Transfusion Sanguine, Paris, France	Ortho Avioq HTLV-I/II Microelisa system	cobas e 411
Nagasaki University Hospital, Nagasaki, Japan	Abbott Architect rHTLV-I/II	cobas e 411
	Fujirebio Lumipulse HTLV-I	
	Fujirebio Serodia-HTLV-I PA	
Roche Diagnostics, Penzberg, Germany	Abbott Architect rHTLV-I/II (performed at Microcoat Biotechnologie GmbH, Bernried, Germany)	Modular Analytics E170
	Abbott Prism HTLV-I/HTLV-II (data provided by sample provider)	cobas e 411
	DiaSorin Murex HTLV I+II	cobas e 601

### Assays.

The Elecsys HTLV-I/II assay (Roche Diagnostics International Ltd., Rotkreuz, Switzerland) is an electrochemiluminescence immunoassay for use on the cobas e analyzers. The three principal comparator screening assays were Ortho Avioq HTLV-I/II Microelisa system (Avioq Inc., Durham, NC, USA), which is an enzyme-linked immunosorbent assay, and Abbott Prism HTLV-I/HTLV-II (Abbott Laboratories, Abbott Park, IL, USA) and Abbott Architect rHTLV-I/II (Abbott Laboratories, Wiesbaden, Germany), which are chemiluminescent immunoassays.

Each laboratory evaluated the Elecsys HTLV-I/II assay and at least one comparator assay (Table 4). All assays were performed according to the manufacturers' instructions. All laboratories used the master pilot lot of the Elecsys HTLV-I/II assay. They were provided with Elecsys HTLV PreciControl and Elecsys HTLV-I/II CalSet (both from single lots) for control and calibration purposes. The confirmatory test used in the specificity evaluation was Fujirebio INNO-LIA HTLV-I/II Score (Fujirebio Diagnostics Inc., Malvern, PA, USA) at all laboratories. In the evaluation of cross-reacting factors, the comparator screening assay at the Penzberg facility was DiaSorin Murex HTLV I+II (DiaSorin, Saluggia, Italy), which is an enzyme-linked immunosorbent assay. Key features of the screening assays are summarized in Table 5.

**TABLE 5:** Key features of the principal HTLV-1/2 screening assays used in the study^*a*^

Assay name (reference[s])	Mfr	Assay type	Analyzer system	Key principle of assay	Testing time (min)
Elecsys HTLV-I/II (19)	Roche Diagnostics	One-step double-antigen sandwich chemiluminescent immunoassay	Modular Analytics E170 or cobas e 411, cobas e 601, cobas e 602	Detects antibodies to viral recombinant antigens gp21 and p24	18
Abbott Architect rHTLV-I/II (21)	Abbott Laboratories	Two-step chemiluminescent immunoassay	Architect *i* system	Detects antibodies to viral synthetic peptide gp46 and recombinant antigen gp21	29
Ortho Avioq HTLV-I/II Microelisa system (24)	Avioq Inc.	ELISA	Microelisa plate reader	Detects antibodies to viral antigens (purified viral lysate) and recombinant HTLV-1 p21E antigen	150
Abbott Prism HTLV-I/HTLV-II (23, 27)	Abbott Laboratories	Three-step sandwich chemiluminescent immunoassay	Abbott Prism system	Detects antibodies to HTLV-1 and HTLV-2 antigens	54
DiaSorin Murex HTLV I+II (28)	DiaSorin	ELISA	Microelisa plate reader	Detects antibodies to HTLV-1 and HTLV-2 antigens	90

a Abbreviations: Mfr, manufacturer; ELISA, enzyme-linked immunosorbent assay.

### Samples used for sensitivity analysis.

Assay sensitivity was evaluated at the Roche Diagnostics facility at Penzberg using frozen serum samples provided by Slieagen, USA; the American Red Cross, USA; Universidad Peruana Cayetano Heredia, Peru; Laboratoire CERBA, France; ProMedDx LLC, USA; Trina Bioreactives AG, Switzerland; and the University of Tokyo, Japan (906 samples). The national reference center for human immunodeficiency virus (HIV), hepatitis B virus (HBV), and hepatitis C virus (HCV) in transfusion (INTS), Paris, France (219 samples), and the laboratory in Barcelona, Spain (24 samples), also analyzed samples. All samples (*n* = 1,149) were previously characterized as positive for HTLV-1/2 (926 HTLV-1 positive, 200 HTLV-2 positive, and 23 HTLV-1/2 positive but not subtyped). Assay sensitivity was calculated as the percentage of precharacterized HTLV-1/2-positive samples identified as positive by the assay.

### Samples used for specificity analysis.

At most laboratories, assay specificity was assessed in fresh serum and EDTA plasma samples provided by the individual laboratory (Barcelona used frozen samples and Nagasaki used frozen and fresh samples). All were leftover samples from routine diagnostic requests and blood donations. Assay specificity was tested in 11,575 samples from blood donors and 2,399 routine diagnostic samples that had previously been identified as negative for HTLV-1/2. Assay specificity was evaluated using blood donor samples at four laboratories (Innsbruck [4,048 samples], Hagen [3,813 samples], Porto [2,166 samples], and Barcelona [1,548 samples]) and routine diagnostic samples at two laboratories (Augsburg [1,500 samples] and Nagasaki [899 samples]). Assay specificity was calculated as the percentage of true HTLV-negative samples that tested negative with the assay.

### Samples used to test for cross-reacting factors.

Assays were also tested for interference using 222 frozen serum samples containing potentially cross-reacting factors. These samples had been characterized previously as positive for hepatitis A virus (HAV), HBV, HCV, HIV, herpes simplex virus, rubella virus, Epstein-Barr virus, Escherichia coli, elevated rheumatoid factor (>200 IU/ml), and various autoimmune disorders. Testing for potential cross-reacting factors was conducted at the Roche Diagnostics facility at Penzberg (122 samples) and at the INTS laboratory in Paris (100 samples).

### Methods and analyses.

For all screening assays, determinations were performed as single measurements. In the evaluation of specificity, samples giving an initial reactive (IR) result were retested in duplicate and were considered to be repeatedly reactive (RR) if either of the retest results had a signal/cutoff (s/co) ratio of ≥1.00. The exception to this was the laboratory at Hagen in Germany, where IR samples were retested with a single determination. All positive samples in the specificity evaluation were subjected to confirmatory testing with HTLV immunoblotting. A sample was considered to be true positive, indeterminate, or true negative depending on the result of the HTLV immunoblot assay.

## Supplementary Material

Supplemental material
