# Supplementary material for: Evaluation of Sensitivity and Specificity Performance of Elecsys HTLV-I/II Assay in a Multicenter Study in Europe and Japan
Source: J Clin Microbiol. 2017 Jun 23;55(7):2180–7. doi: 10.1128/JCM.00169-17 (PMC5483920; doi:10.1128/JCM.00169-17)
Supplement: Supplemental material [file supp_55_7_2180__index.html]

Supplemental material 

# Evaluation of Sensitivity and Specificity Performance of Elecsys HTLV-I/II Assay in a Multicenter Study in Europe and Japan

## Supplemental material

- Supplemental file 1 -

  Tables S1 (Specificity analyses for the Elecsys HTLV-I/II assay and comparator assays in blood donor samples at individual laboratories) and S2 (Specificity analyses for the Elecsys HTLV-I/II assay and comparator assays in routine diagnostic samples at individual laboratories)

  PDF, 115K
