# Supplementary material for: Evaluation of Sensitivity and Specificity Performance of Elecsys HTLV-I/II Assay in a Multicenter Study in Europe and Japan
Source: J Clin Microbiol. 2017 Jun 23;55(7):2180–7. doi: 10.1128/JCM.00169-17 (PMC5483920; doi:10.1128/JCM.00169-17)
Supplement: Supplemental material [file JCM.00169-17_zjm999095567s1.pdf]

SUPPLEMENTAL MATERIAL

TABLE S1 Specificity analyses for the Elecsys® HTLV-I/II assay and comparator assays in blood donor samples at individual laboratories

Innsbruck, Austria

MODULAR® *ANALYTICS* E170	Elecsys® HTLV-I/II Serum	Elecsys® HTLV-I/II Plasma	Abbott Architect® rHTLV-I/II Serum	Ortho Avioq® HTLV-I/II Serum
n total	2024	2024	2024	2024
**n negative**	**2022**	**2022**	**2022**	**2022**
IRpos ≥1 s/co	2	1	0	3
IRfpos ≥1 s/co	1	0	0	2
RRpos ≥1 s/co	2	1	0	2
RRfpos ≥1 s/co	1	0	0	1
Immunoblot positive HTLV-I/II	0/2	0/1	0/0	0/3
Immunoblot indeterminate HTLV-I/II	1/2	1/1	0/0	1/3
Immunoblot negative HTLV-I/II	1/2	0/1	0/0	2/3

Specificity % IR ≥1 s/co	99.95	100	100	99.90
Confidence limit % (95%; 2-sided. IR ≥1)	99.73–100	99.82–100	99.82–100	99.64–99.99
**Specificity % RR ≥1 s/co**	**99.95**	**100**	**100**	**99.95**
Confidence limit % (95%; 2-sided. RR ≥1)	99.73–100	99.82–100	99.82–100	99.72–100

n total, number of analyzed samples; n negative, number of true-negative samples (excluding indeterminate, positive, and false-negative samples); IR, initially reactive; IRpos, initially reactive sample, including true- positive samples; IRfpos, initially reactive sample, not including true-positive samples; RR, repeatedly reactive; RRpos, repeatedly reactive sample, including true-positive samples; RRfpos, repeatedly reactive sample, not including true-positive samples; s/co, signal/cutoff.

Hagen, Germany

**cobas e** 602	Elecsys® HTLV-I/II Serum	Abbott Prism® HTLV-I/HTLV-II Serum
n total	3813	3813
**n negative**	**3812**	**3812**
IRpos ≥1 s/co	2	5
IRfpos ≥1 s/co	1	5
RRpos ≥1 s/co	2	0
RRfpos ≥1 s/co	1	0
Immunoblot positive HTLV-I/II	0/2	0/0
Immunoblot indeterminate HTLV-I/II	1/2	0/0
Immunoblot negative HTLV-I/II	1/2	0/0
Specificity % IR ≥1 s/co	99.97	99.87
Confidence limit % (95%; 2-sided. IR ≥1)	99.85–100	99.69–99.96
**Specificity % RR ≥1 s/co**	**99.97**	**100**
Confidence limit % (95%; 2-sided. RR ≥1)	99.85–100	99.90–100

n total, number of analyzed samples; n negative, number of true-negative samples (excluding indeterminate, positive, and false-negative samples); IR,

initially reactive; IRpos, initially reactive sample, including true-positive samples; IRfpos, initially reactive sample, not including true-positive samples; RR, repeatedly reactive; RRpos, repeatedly reactive sample, including true-positive samples; RRfpos, repeatedly reactive sample, not including true-positive samples; s/co, signal/cutoff.

Porto, Portugal

**cobas e** 601	Elecsys® HTLV-I/II Serum	Abbott Architect® rHTLV-I/II Serum
n total	2166	2166
**n negative**	**2166**	**2166**
IRpos ≥1 s/co	2	2
IRfpos ≥1 s/co	2	2
RRpos ≥1 s/co	2	2
RRfpos ≥1 s/co	2	2
Immunoblot positive HTLV-I/II	0/2	0/2
Immunoblot indeterminate HTLV-I/II	0/2	0/2
Immunoblot negative HTLV-I/II	2/2	2/2
Specificity % IR ≥1 s/co	99.91	99.91
Confidence limit % (95%; 2-sided. IR ≥1)	99.67–99.99	99.67–99.99
**Specificity % RR ≥1 s/co**	**99.91**	**99.91**
Confidence limit % (95%; 2-sided. RR ≥1)	99.67–99.99	99.67–99.99

n total, number of analyzed samples; n negative, number of true-negative samples (excluding indeterminate, positive, and false-negative samples); IR,

initially reactive; IRpos, initially reactive sample, including true-positive samples; IRfpos, initially reactive sample, not including true-positive samples; RR, repeatedly reactive; RRpos, repeatedly reactive sample, including true-positive samples; RRfpos, repeatedly reactive sample, not including true-positive samples; s/co, signal/cutoff.

Barcelona, Spain

**cobas e** 411	Elecsys® HTLV-I/II Serum	Abbott Architect® rHTLV-I/II Serum
n total	1547*	1547*
**n negative**	**1546**	**1546**
IRpos ≥1 s/co	2	4
IRfpos ≥1 s/co	2	3
RRpos ≥1 s/co	2	4
RRfpos ≥1 s/co	2	3
Immunoblot positive HTLV-I/II	0/2	0/4
Immunoblot indeterminate HTLV-I/II	0/2	1/4
Immunoblot negative HTLV-I/II	2/2	3/4
Specificity % IR ≥1 s/co	99.87	99.81
Confidence limit % (95%; 2-sided. IR ≥1)	99.53–99.98	99.43–99.96
**Specificity % RR ≥1 s/co**	**99.87**	**99.81**
Confidence limit % (95%; 2-sided. RR ≥1)	99.53–99.98	99.43–99.96

* One sample excluded from specificity calculations:

Elecsys® HTLV-I/II reactive; Abbott Architect® rHTLV-I/II nonreactive; Fujirebio INNO-LIA® HTLV I/II Score reactive: "env gp21 I/II"("1+"), "env gp46 I/II", "env gp46-I" ("+/-"); no clarification by nucleic acid technology (NAT) testing or by a sequential bleed was possible due to anonymization of samples.

n total, number of analyzed samples; n negative, number of true-negative samples (excluding indeterminate, positive, and false-negative samples); IR, initially reactive; IRpos, initially reactive sample, including true-positive samples; IRfpos, initially reactive sample, not including true-positive samples; RR, repeatedly reactive; RRpos, repeatedly reactive sample, including true-positive samples; RRfpos, repeatedly reactive sample, not including true-positive samples; s/co, signal/cutoff.

TABLE S2 Specificity analyses for the Elecsys® HTLV-I/II assay and comparator assays in routine diagnostic samples at individual laboratories

Augsburg, Germany (request on hepatitis/HIV testing and pregnant women): overall results

MODULAR® *ANALYTICS* E170	Elecsys® HTLV-I/II Serum	Abbott Architect® rHTLV-I/II Serum
n total	1500	1500
**n negative**	**1499**	**1499**
IRpos ≥1 s/co	5	7
IRfpos ≥1 s/co	4	6
RRpos ≥1 s/co	5	7
RRfpos ≥1 s/co	4	6
Immunoblot positive HTLV-I/II	0/5	0/7
Immunoblot indeterminate HTLV-I/II	1/5	1/7
Immunoblot negative HTLV-I/II	4/5	6/7
Specificity % IR ≥1 s/co	99.73	99.60
Confidence limit % (95%; 2-sided. IR ≥1)	99.32–99.93	99.13–99.85
**Specificity % RR ≥1 s/co**	**99.73**	**99.60**

Confidence limit % (95%; 2-sided. RR ≥1)	99.32–99.93	99.13–99.85

n total, number of analyzed samples; n negative, number of true-negative samples (excluding indeterminate, positive, and false-negative samples); IR, initially reactive; IRpos, initially reactive sample, including true-positive samples; IRfpos, initially reactive sample, not including true-positive samples; RR, repeatedly reactive; RRpos, repeatedly reactive sample, including true-positive samples; RRfpos, repeatedly reactive sample, not including true-positive samples; s/co, signal/cutoff.

Augsburg, Germany (request on hepatitis/HIV testing)

MODULAR® *ANALYTICS* E170	Elecsys® HTLV-I/II Serum	Abbott Architect® rHTLV-I/II Serum
n total	500	500
**n negative**	**500**	**500**
IRpos ≥1 s/co	2	3
IRfpos ≥1 s/co	2	3
RRpos ≥1 s/co	2	3
RRfpos ≥1 s/co	2	3
Immunoblot positive HTLV-I/II	0/2	0/3
Immunoblot indeterminate HTLV-I/II	0/2	0/3
Immunoblot negative HTLV-I/II	2/2	3/3
Specificity % IR ≥1 s/co	99.60	99.40
Confidence limit % (95%; 2-sided. IR ≥1)	99.56–99.95	99.26–99.88
**Specificity % RR ≥1 s/co**	**99.60**	**99.40**
Confidence limit % (95%; 2-sided. RR ≥1)	99.56–99.95	99.26–99.88

n total, number of analyzed samples; n negative, number of true-negative samples (excluding indeterminate, positive, and false-negative samples); IR,

initially reactive; IRpos, initially reactive sample, including true-positive samples; IRfpos, initially reactive sample, not including true-positive samples; RR, repeatedly reactive; RRpos, repeatedly reactive sample, including true-positive samples; RRfpos, repeatedly reactive sample, not including true-positive samples; s/co, signal/cutoff.

Augsburg, Germany (pregnant women)

MODULAR® *ANALYTICS* E170	Elecsys® HTLV-I/II Serum	Abbott Architect® rHTLV-I/II Serum
n total	1000	1000
**n negative**	**999**	**999**
IRpos ≥1 s/co	3	4
IRfpos ≥1 s/co	2	3
RRpos ≥1 s/co	3	4
RRfpos ≥1 s/co	2	3
Immunoblot positive HTLV-I/II	0/3	0/4
Immunoblot indeterminate HTLV-I/II	1/3	1/4
Immunoblot negative HTLV-I/II	2/3	3/4
Specificity % IR ≥1 s/co	99.80	99.70
Confidence limit % (95%; 2-sided. IR ≥1)	99.28–99.98	99.12–99.94
**Specificity % RR ≥1 s/co**	**99.80**	**99.70**
Confidence limit % (95%; 2-sided. RR ≥1)	99.28–99.98	99.12–99.94

n total, number of analyzed samples; n negative, number of true-negative samples (excluding indeterminate, positive, and false-negative samples); IR,

initially reactive; IRpos, initially reactive sample, including true-positive samples; IRfpos, initially reactive sample, not including true-positive samples; RR, repeatedly reactive; RRpos, repeatedly reactive sample, including true-positive samples; RRfpos, repeatedly reactive sample, not including true-positive samples; s/co, signal/cutoff.

Nagasaki, Japan

**cobas e** 411	Elecsys® HTLV-I/II Serum	Abbott Architect® rHTLV-I/II Serum	Fujirebio Lumipulse® HTLV-I Serum	Fujirebio Serodia® HTLV-I Serum
n total	899	899	899	899
**n negative**	**837**	**837**	**837**	**837**
IRpos ≥1 s/co	61	61	62	59
IRfpos ≥1 s/co	0	1	1	0
RRpos ≥1 s/co	61	61*	na	na
RRfpos ≥1 s/co	0	1	na	na
Immunoblot positive HTLV-I/II	59/61	59/61	59/62	59/59
Immunoblot indeterminate HTLV-I/II	2/61	1/61	2/62	0/59
Immunoblot negative HTLV-I/II	0/61	1/61	1/62	0/59
Specificity % IR ≥1 s/co	100	99.88	99.88	100
Confidence limit % (95%; 2-sided. IR ≥1)	99.56–100	99.34–100	99.34–100	99.56–100

**Specificity % RR ≥1 s/co**	**100**	**99.88**	**na**	**na**
Confidence limit % (95%; 2-sided. RR ≥1)	99.56–100	99.34–100	na	na

* One sample was initially reactive but no material was available for repetition – the sample was counted as RRpos because immunoblot was positive.

n total, number of analyzed samples; n negative, number of true-negative samples (excluding indeterminate, positive, and false-negative samples); na, not applicable; IR, initially reactive; IRpos, initially reactive sample, including true- positive samples; IRfpos, initially reactive sample, not including true-positive samples; RR, repeatedly reactive; RRpos, repeatedly reactive sample, including true-positive samples; RRfpos, repeatedly reactive sample, not including true-positive samples; s/co, signal/cutoff.
